# Supplementary material for: Automated highly multiplexed super-resolution imaging of protein nano-architecture in cells and tissues
Source: Nat Commun. 2020 Mar 25;11:1552. doi: 10.1038/s41467-020-15362-1 (PMC7096454; doi:10.1038/s41467-020-15362-1)
Supplement: Supplementary file 1 — Supplementary Information [file 41467_2020_15362_MOESM1_ESM.pdf]

SUPPLEMENTARY INFORMATION

Automated highly multiplexed super-resolution imaging of protein nano-architecture in cells and tissues

Klevanski et al.

Supplementary information guide

Supplementary Element	Title	Location
Supplementary Figure 1 with legend	Microscope components of the maS^{3}TORM setup	SI file, p. 3
Supplementary Figure 2 with legend	Software controlling the maS^{3}TORM setup	SI file, p. 4
Supplementary Figure 3 with legend	Graphical user interface (GUI) of the Experiment Editor software	SI file, pp. 5-6
Supplementary Figure 4 with legend	Communication between the microscope control and the robot software	SI file, pp. 7-8
Supplementary Figure 5 with legend	Control experiments for different bleaching and elution conditions	SI file, p. 9
Supplementary Figure 6 with legend	Control experiments for bleaching- and elution-mediated signal removal	SI file, pp. 10-11
Supplementary Figure 7 with legend	Representative images for control experiments of bleaching- and elution-mediated signal removal	SI file, p. 12
Supplementary Figure 8 with legend	Single case control experiments for estimation of cross-talk and labeling efficiency	SI file, pp. 13-14
Supplementary Figure 9 with legend	Signal preservation and structural integrity during repeated bleaching and elution	SI file, pp. 15-16
Supplementary Figure 10 with legend	Localization precision analysis for images acquired throughout the multiplex experiment	SI file, pp. 17-18
Supplementary Figure 11 with legend	Examples showing 3D visualization of selected STORM images	SI file, p. 19
Supplementary Figure 12 with legend	Workflow for analysis of presynaptic architecture of the calyx of Held	SI file, pp. 20-21
Supplementary Figure 13 with legend	Averaged line profiles of global and active zone-specific protein distributions	SI file, p. 22
Supplementary Figure 14 with legend	Colocalization matrix. Pearson's r values for the colocalization matrix shown in Figure 3m	SI file, p. 23
Supplementary Note 1	Extended information for control experiments for signal removal (Supplementary Fig. 6a)	SI file, pp. 24-25
Supplementary Note 2	Set of rules helping the user to design an optimal multiplex experiment	SI file, p. 25
Supplementary Note 3	Supplementary discussion of registration precision between different staining rounds for cells or tissue	SI file, p. 26
References	Supplementary references	SI file, p. 27
Supplementary Table 1	Experimental workflow for multiplex experiment in U2OS cells shown in Figure 2a	SI file, p. 28
Supplementary Table 2	Experimental workflow for the multiplex experiment in the medial nucleus of the trapezoid body shown in Figure 3c	SI file, p. 29
Supplementary Table 3	All antibodies and other labels used in this work	SI file, p. 30
Supplementary Table 4	maS^{3}TORM components	SI file, p. 31
Supplementary Table 5	Exact number of experiments, samples, and selections for all quantified analyses	SI file, p. 32
Supplementary Video 1	Performance of the maS^{3}TORM setup	online
Supplementary Video 2	Super-resolution images of three target proteins from different imaging rounds merged in 3D space	online

SI = Supplementary Information

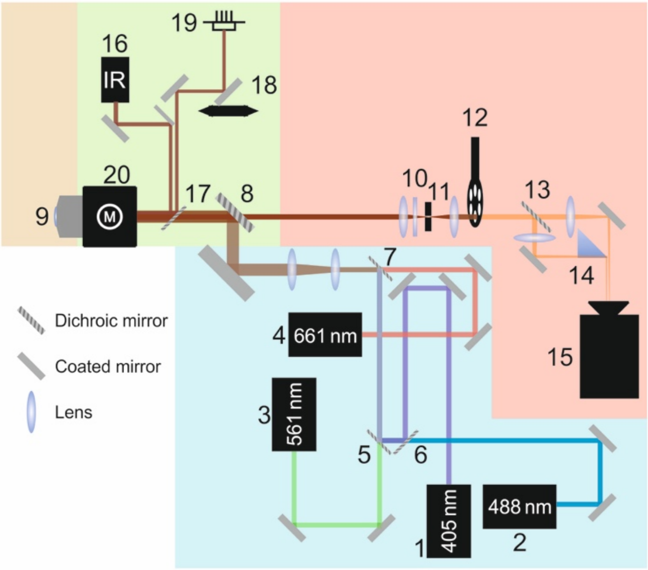

Supplementary Figure 1 | Microscope components of the maS^{3}TORM setup. Schematic of the microscope components of the maS^{3}TORM setup (for a list of components see **Supplementary Table 4**). For emission (blue background), the home-built *d*STORM system is equipped with four lasers: 405 nm (#1), 488 nm (#2), 561 nm (#3), and 661 nm (#4). The 405 nm, 488 nm, and the 561 nm laser beams are merged using two dichroic mirrors (#5 and #6). The 661 nm laser beam that is primarily used for *d*STORM measurements is conjoined with all the other laser beams by the dichroic mirror (#7). Subsequently, the excitation light is widened by two lenses forming a telescope and is reflected onto the sample using another dichroic mirror (#8). The emission beam (red background) is collected by the objective (#9; yellow background) and passes a tube lens followed by the cylindrical lens (#10) that can be used for 3D imaging. At the focal point of the tube lens a slit (#11) is placed, followed by another lens parallelizing the beam. Different emission filters to block excitation light are mounted in a filter wheel (#12). The dichroic filter (#13) splits the emission light into two beams with one beam passing and the other being reflected at the inner edge of a prism (#14) resulting in two channels that are focused onto the camera (#15). The microscope is further provided with an infrared beam-based active focus stabilization system (green background). The incoming beam of the infrared diode (#16) is coupled into the objective by the dichroic mirror (#17). The beam is reflected at the edge between the glass surface of the sample and the sample medium. Using a movable mirror (#18), the reflected beam is projected onto a quadrant diode (#19). To correct for focus drift, the quadrant diode is linked to a controller (not shown) that, in turn, initiates movement of a piezo-driven stage (#20) wherein the objective is mounted.

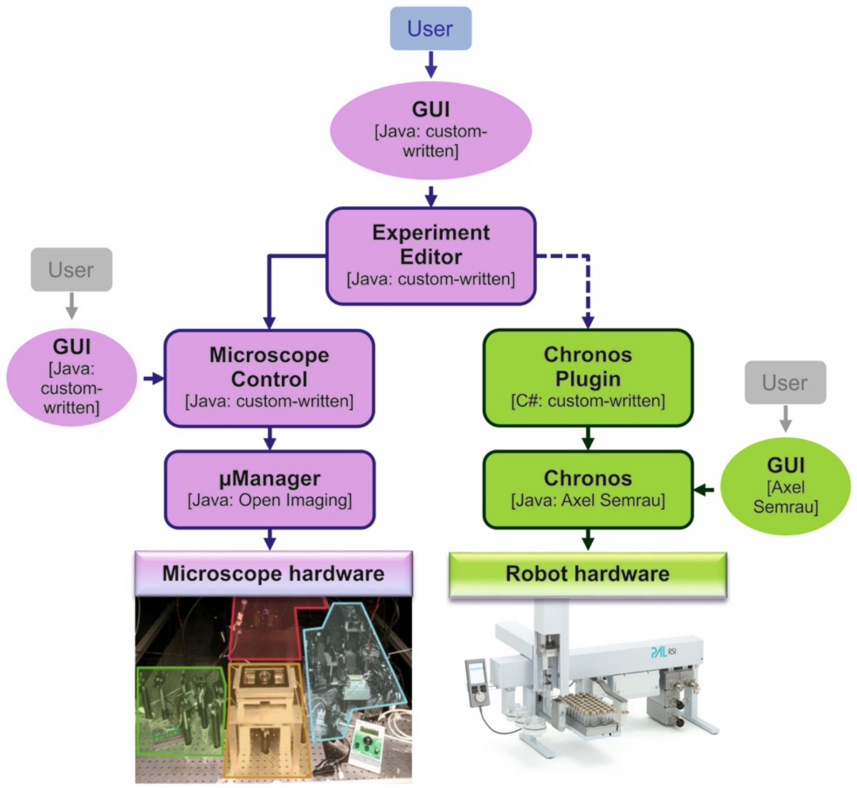

Supplementary Figure 2 | Software controlling the maS^{3}TORM setup. All microscope hardware components (photograph at the left lower part of the diagram) were implemented using the open-source software µManager[1]. A custom-written Microscope Control software featuring a graphical user interface (GUI) was developed to conveniently use all necessary functionality of the hardware. The setup was complemented by the commercial pipetting robot PAL3 by CTC Analytics AG/Axel Semrau GmbH & Co KG (photograph at the lower right provided by CTC Analytics AG with permission to publish) and can be controlled by Chronos software supplied by the manufacturer (Axel Semrau GmbH & Co KG). For a fully automated microscopy and re-staining procedure, both microscope hardware elements and the pipetting robot were integrated into a custom-written Experiment Editor software that can operated via a GUI. While the Microscope Control software is directly addressed by the Experiment Editor software, the communication between Experiment Editor and the pipetting robot is realized indirectly (depicted by the dashed line) via an exchange folder (see **Supplementary Figure 4** for more details). To ensure seamless communication, the folder is continuously checked and modified by the custom-written plugin for Chronos as well as by the Experiment Editor.

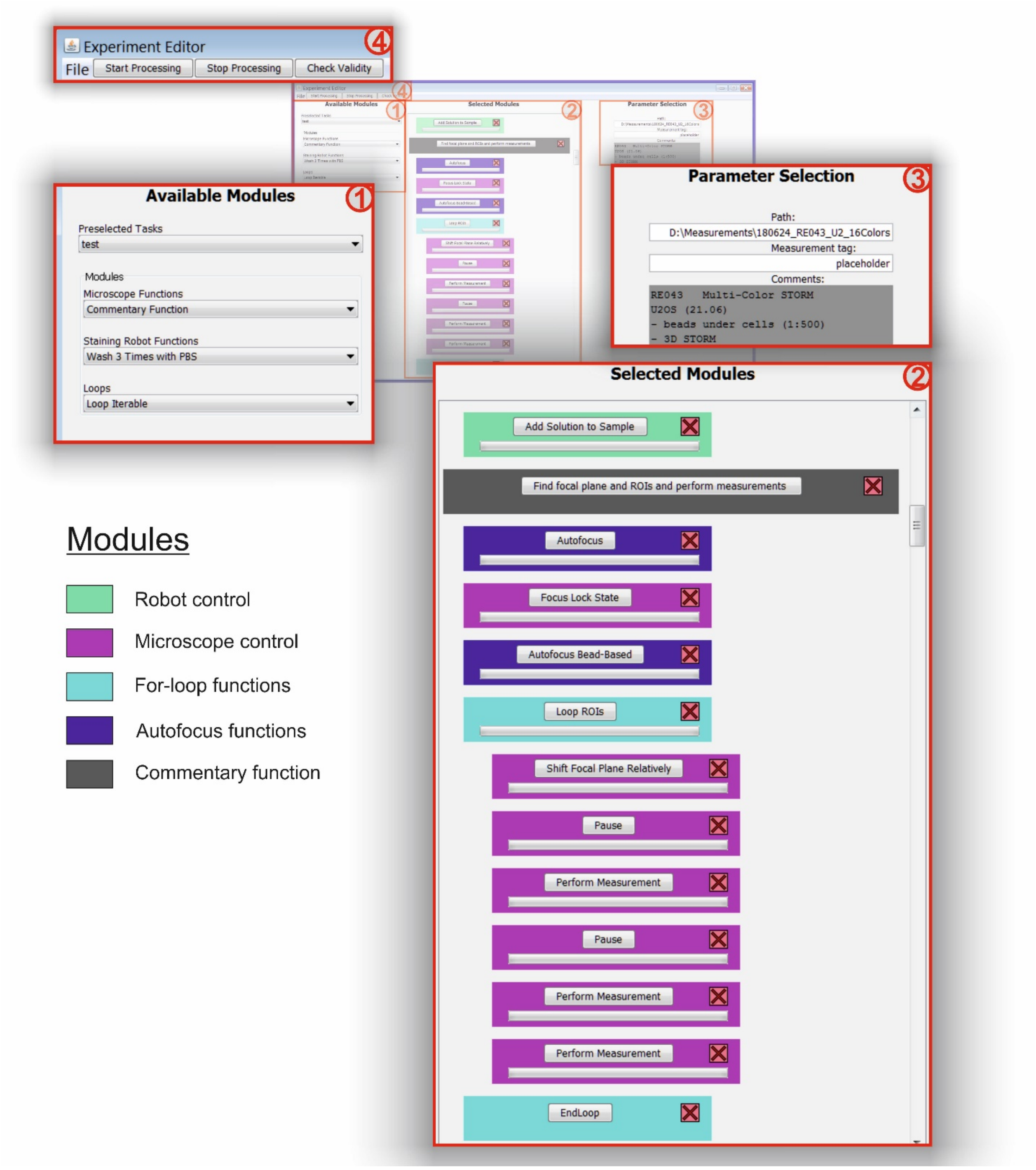

Supplementary Figure 3 | Graphical user interface (GUI) of the Experiment Editor software. To create a customized experiment, the user can select modules from three main categories listed in the 'Available Module' section (1). The 'Microscope Functions' dropdown list contains all modules that directly control the hardware of the microscope such as lasers, stage, or camera. The 'Staining Robot Functions' list contains modules that trigger different actions of the robot. Most modules bundle different robot actions together for a convenient and time-saving usage. The 'Loops' dropdown category contains two loop modules: 'Loop Iterable' and 'Loop ROIs'. 'Loop Iterable' is used to repeat modules of choice for a desired number of times while 'Loop ROIs' is designed to repeat microscopic actions for a list of chosen ROIs that are automatically approached by the microscope stage. Once a module is chosen from

one of the three categories, it appears in the 'Selected Modules' column in the center of the GUI (2). An exemplary excerpt of a typical experiment is shown. It contains all types of modules: robot control-related (green), microscope control-related (magenta), and loop functions (light blue). In addition, autofocus functions (dark blue) and the commentary function (gray) can be selected from the 'Microscope Control' list. After being added to the 'Selected Modules' field, the order of all modules can be changed by the drag and drop principle. By clicking the cross on red background, modules can be deleted from the list. Gray push-buttons of individual modules can be clicked to display and modify module-specific parameters, which are shown in the right 'Parameter Selection' part of the GUI (3). After an experiment is designed, its consistency can be checked by the 'Check Validity' button on the upper left corner of the GUI (4). This function performs simple checks to ensure that, for example, each parameter field is filled and that loops have the right number of parameters corresponding to the number of repetitions. Subsequently, the workflow can be executed by clicking the 'Start Processing' and interrupted by the 'Stop Processing' button. The 'File' menu provides possibilities to save and load workflows.

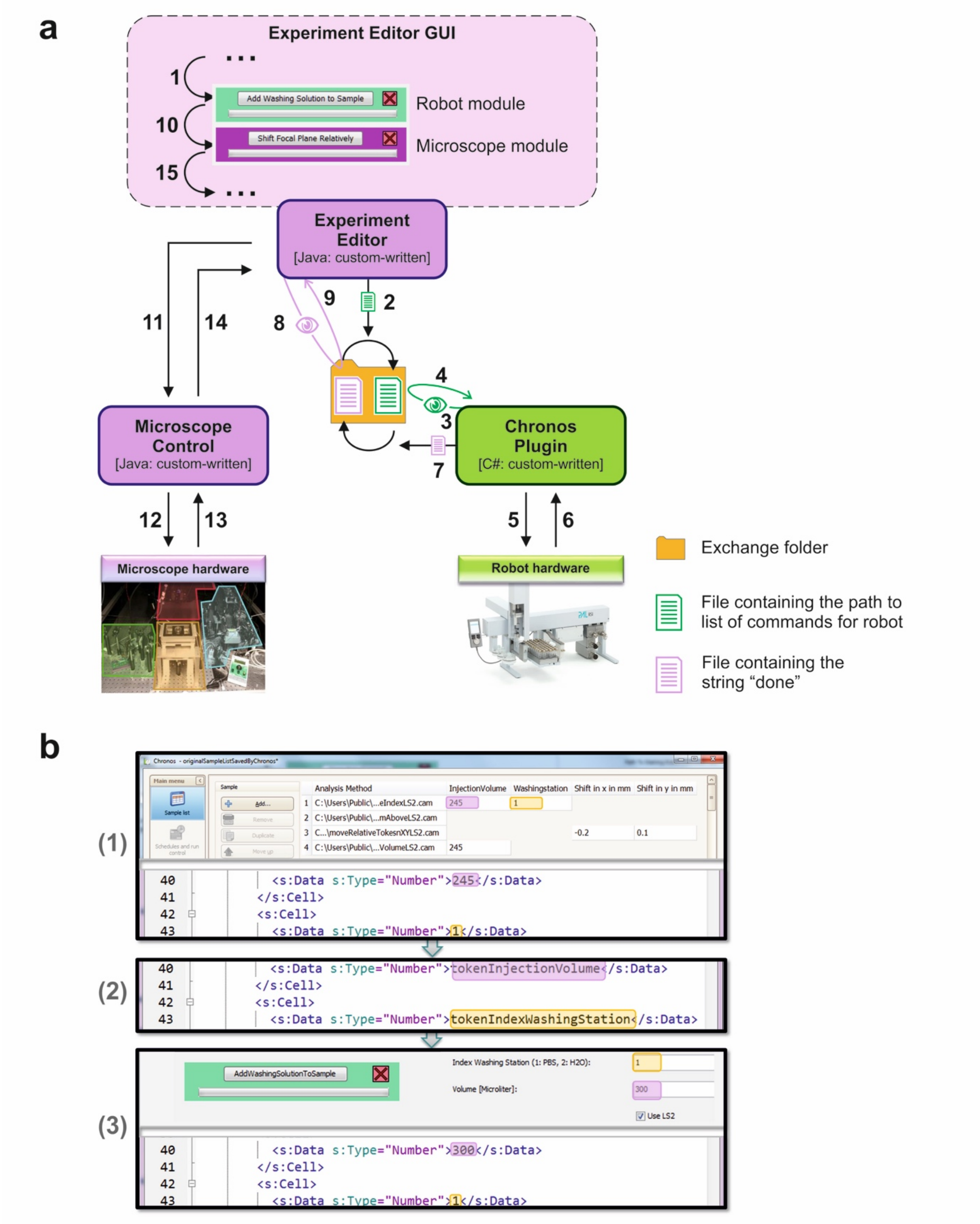

Supplementary Figure 4 | Communication between the microscope control and the robot software. (**a**) After launching a robot module in the Experiment Editor (1), a text file containing the path to a list of specific robot commands is saved to the exchange folder (2). The Chronos plugin continuously checks for the text file. Once the desired file has been found (3), the path is opened and the respective list of commands is imported in Chronos (4) and executed by the robot (5; photograph at the lower right provided by CTC Analytics AG with permission to publish). After successful execution, Chronos receives a response (6) and the plugin saves a file containing the string “done” to the exchange folder (7). The Experiment

Editor monitors the exchange folder (8), finds the “done” file (9), and jumps to the next module (10). If the next module is a microscope-related module, the command is transferred to the Microscope Control software (11) and to the hardware components of the microscope (12). After the command has been executed, a feedback is given to the Microscope Control (13) and to the Experiment Editor (14) that proceeds to the next module (15). (**b**) Experiment Editor modules for the robot ensure maximal flexibility by using variables. In this example the preparation of a simple module for the addition of variable volume (magenta highlighted fields) of solutions from variable washing stations (yellow highlighted fields) to the sample (see upper part of panel 3) is described. First, within the Chronos software (Screenshot with permission of Axel Semrau GmbH & Co KG) a list of commands called “Sample list”, in turn, consisting of smaller command blocks called “Analysis methods” is created (upper part of panel 1) and saved as a command list template in the cml format (lower part of panel 1). Second, values that are intended to be specified within the Experiment Editor GUI are substituted by specific variables (panel 2). Third, when the robot-related module gets executed, the variables in the pre-generated cml template file are automatically substituted by the parameters specified by the user (panel 3) and saved. A file containing the path to the customized command list is saved in the exchange folder as described in (a).

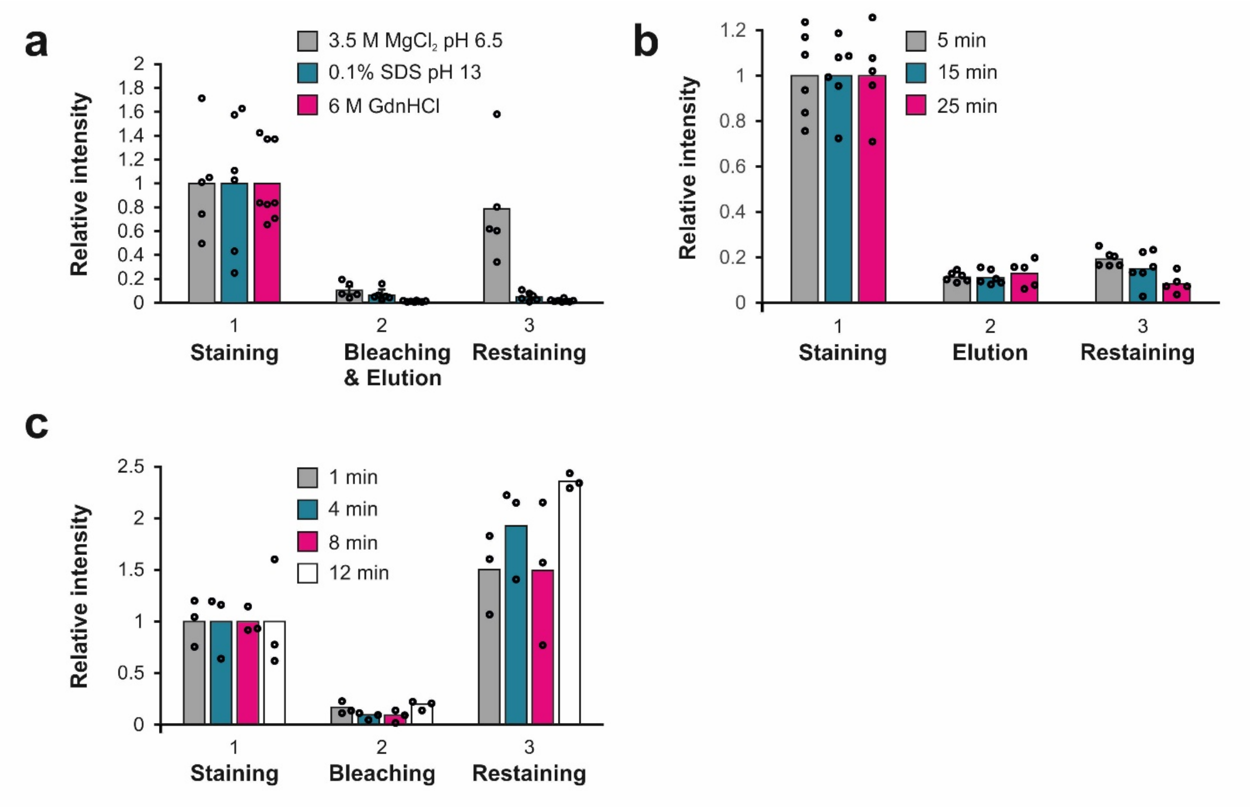

Supplementary Figure 5 | Defining bleaching and elution conditions. For the following control experiments, U2OS cells were initially (before the 1st imaging round) stained with primary antibodies against Tom20 (from rabbit) and Alexa 647 labeled secondary antibodies against rabbit. The 2nd imaging round was performed after elution/and or bleaching and the 3rd round after cells were re-stained with secondary antibody against rabbit. (**a**) Comparison of different elution buffers. Tom20 signal in the initial imaging round; signal remaining after bleaching and treatment with three buffers in the second round; signal that can be retrieved after re-staining. The latter shows that treatment with 3.5 M magnesium chloride elution buffer (Yi et al. (2016)[2]) resulted in the highest cross-talk. Treatment with 0.1% SDS pH 13[3] and 6 M guanidinium hydrochloride (GdnHCl) buffer[4] eluted with similar efficiencies of ~5% and ~1.5%, respectively. Yet, in our hands, GdnHCl strongly impaired the structural integrity of the probe. Thus, we applied the SDS buffer in all further experiments. (**b**) Three different incubation times (5, 15 and 25 min) for elution. Elution performed for 25 min was most efficient, but often resulted in cell detachment (data not shown). To maintain cell attachment, minimize structure damage, and reduce time for multiplex experiments, we performed our experiments with an exposure time of 15 min. (**c**) Four different bleaching times (1, 4, 8, and 12 min) did not differ in efficiency. To ensure efficient bleaching, minimize the effect of photounbinding (see **Supplementary Figure 6**) and minimize time required for multiplex experiments, we chose a bleaching duration of 4 min. (**a-c**) For the full-frame analysis of data, rendered images were split into foreground (Tom20 signal) and background pixels using Ilastic software. Foreground areas were exported as binary masks and loaded in ImageJ together with the rendered images. The average foreground pixel intensity per signal-positive area was calculated using a custom-written script in ImageJ. Bars represent mean values. For sample numbers, see **Supplementary Table 5**. Source data are provided as a Source Data file.

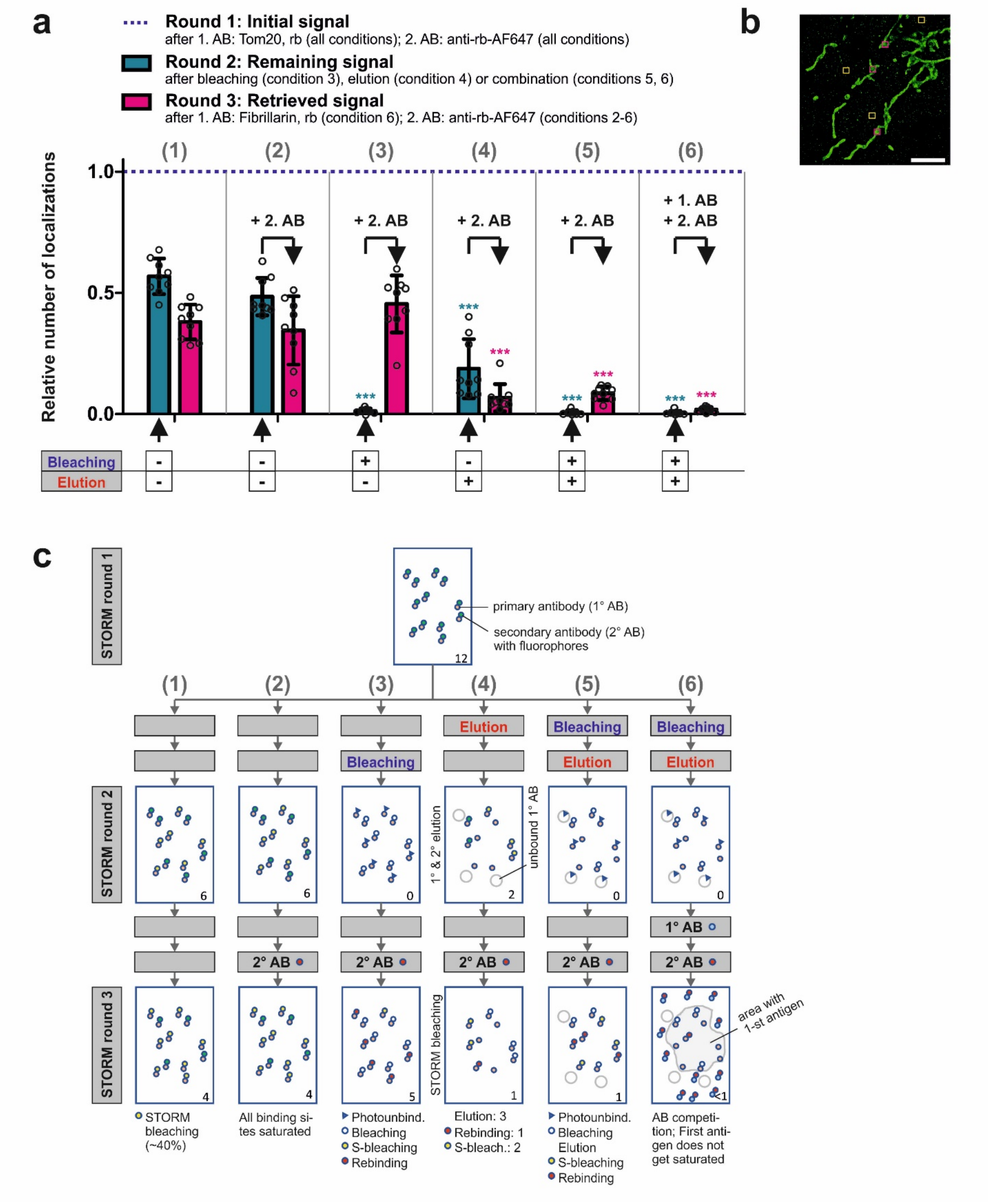

Supplementary Figure 6 | Control experiments for bleaching- and elution-mediated signal removal. (**a**) Six test experiments evaluating the cross-talk between consecutive multiplexed imaging rounds. U2OS cells were stained with a primary antibody against Tom20 from rabbit (rb) and a secondary anti-rabbit antibody conjugated to Alexa Fluor 647. Three STORM imaging rounds were carried out for each condition, see **Supplementary Note 1** for a detailed description. The dashed line reflects the average signal intensity normalized to the initial staining (obtained by quantifying localizations within selected Tom20-positive regions).

Bleaching was either induced by the STORM acquisition itself or by the intended photobleaching procedure (see Methods). The secondary antibody was reapplied and the percentage of signal retrieval was evaluated. To measure the influence of a competing primary antibody (test condition 6), rabbit Fibrillarin antibody was applied. Each experiment was carried out for 3 independent sample dishes with 3 cells analyzed per dish. For Tom20 signal quantifications, 3 rectangular selections in mitochondria-positive areas were analyzed per cell (b). Background signal was determined in cell area lacking mitochondria (3 selections/cell) and subtracted from Tom20 signal to quantify specific Tom20 signal. Bars represent mean ± SD. For statistical analysis, all bars corresponding to imaging round 2 were compared to round 2 of the first test condition. In analogy, all bars corresponding to imaging round 3 were compared to round 3 from the first test condition. Asterisks indicate statistical significance (*** corresponding to $p < 0.001$), assessed by one-way ANOVA followed by Bonferroni post-hoc test. (**b**) Example showing how rectangular selections (8.8 nm×8 nm) were placed in Tom20-positive areas (magenta) and background regions (yellow) to quantify the number of localizations. As only selections *completely* covered by Tom20 signal were used for the analysis, no normalization to surface area was necessary. Scale bar 5 µm. (**c**) Graphical representation of the experiment shown in (a). Exemplary numbers of antigens labeled by antibodies with intact fluorophores are shown in the right bottom corner of each panel illustrating an imaging round. Source data, including exact p values, are provided as a Source Data file.

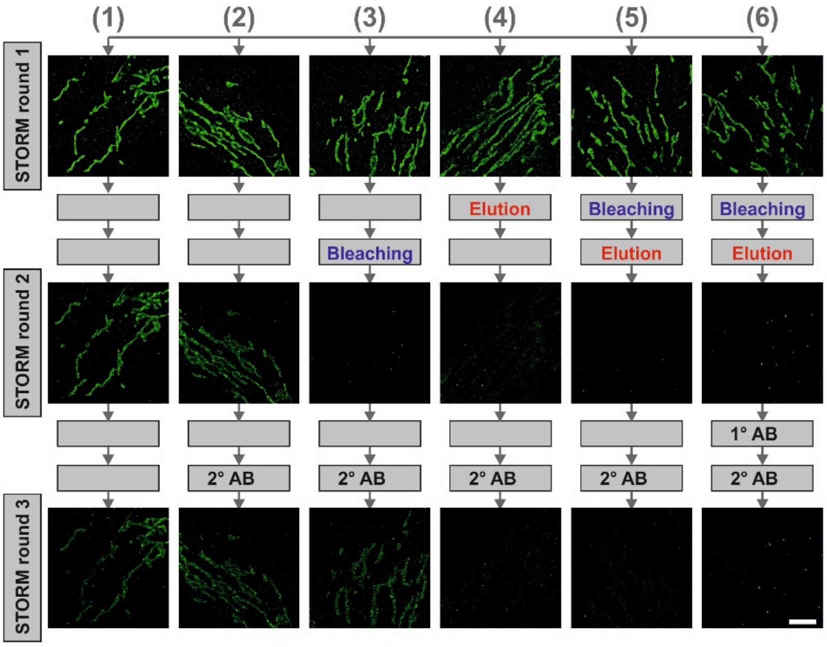

Supplementary Figure 7 | Representative images for control experiments of bleaching- and elution-mediated signal removal. Representative images showing how Tom20 signal appeared during the six different test conditions presented in **Supplementary Figure 6a**.

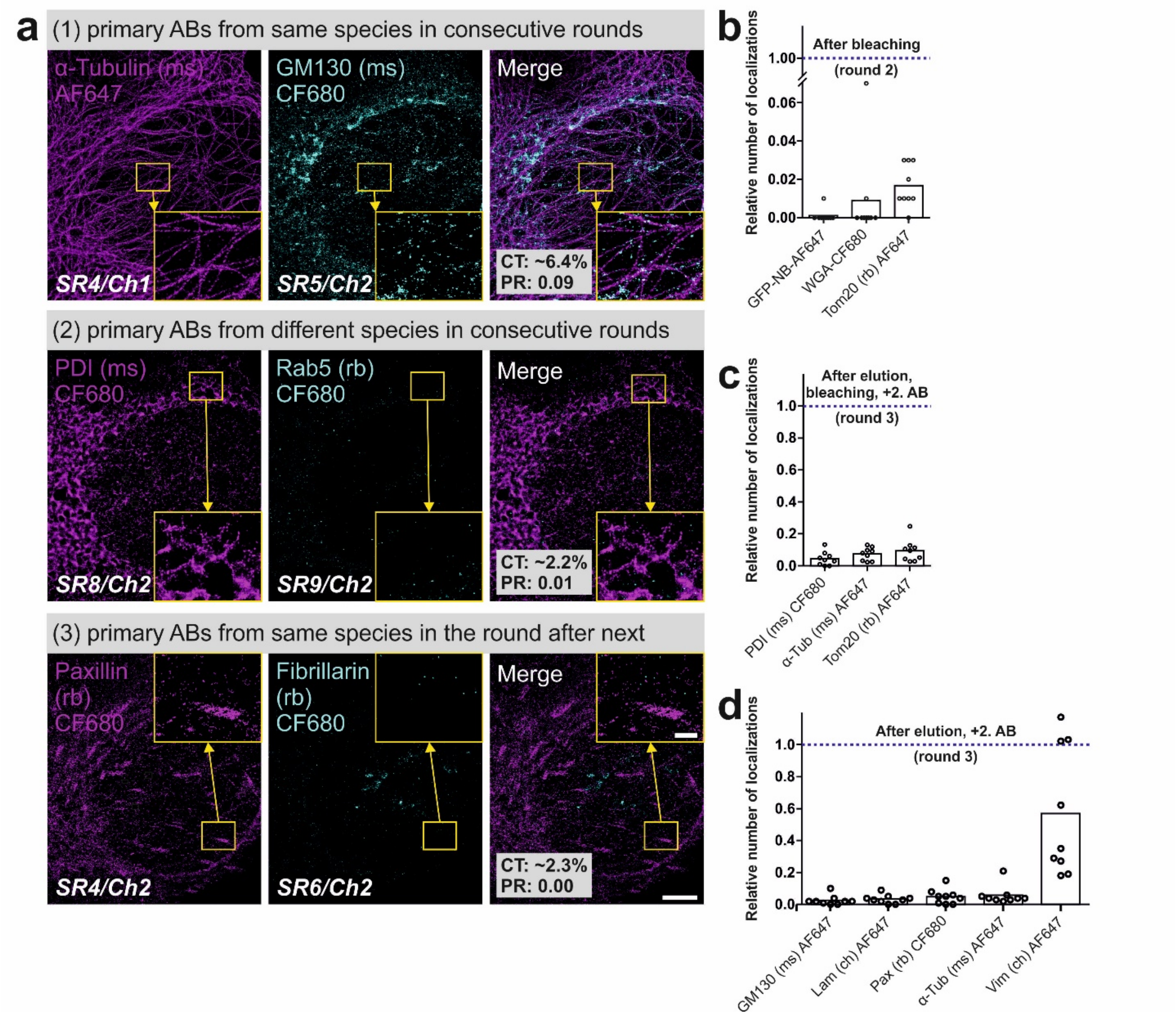

Supplementary Figure 8 | Single case control experiments for estimation of cross-talk and labeling efficiency. (**a**) Three examples taken from **Figure 2a** were analyzed with respect to cross-talk from preceding staining rounds (SRs). Row 1: consecutive rounds employing primary antibodies from the same species (mouse (ms)) show a cross-talk (CT) of ~6.4% between α-tubulin and GM130 channels (Ch). Colocalization analysis revealed a low Pearson's r (PR) coefficient of 0.09. Row 2: Primary antibodies of different species (ms and rabbit (rb)), ~2.2% CT and 0.01 PR. Row 3: CT ~ 2.3% and PR = 0 when using primary antibodies from same species in the next but one round. CT was calculated using five rectangular selections (8.8 nm×8 nm) in tubulin-positive areas containing ≥ 3 strands, Golgi-negative areas, in PDI-positive areas, and in Paxilin-positive areas (avoiding nucleoli). Five selections devoid of any specific signal defined background. CT value was calculated from ratio of background-corrected signals determined in earlier and later rounds. PR was quantified in full-size images using Image J Coloc 2 function. CT and PR coefficients might be overestimated because unknown ground truth of the second structure may include regions overlapping with the previous round. Scale bars 5 μm in main panels and 1 μm in magnified boxed areas. (**b**) The effect of bleaching in consecutive rounds in three examples yielding very low CT values of ~0.1% to ~1.7%. (**c**) Combination of elution and bleaching with subsequent reapplication of the same secondary antibody (2˚ AB) resulting in CT values ranging from 4.5% to 9.3%. (**d**) Effect of elution (without the additional bleaching) evaluated in five examples

resulting in values below 6% for most test cases. The very high affinity of the vimentin antibody resulted in an unusually high CT. For sample numbers, see **Supplementary Table 5**. Source data are provided as a Source Data file.

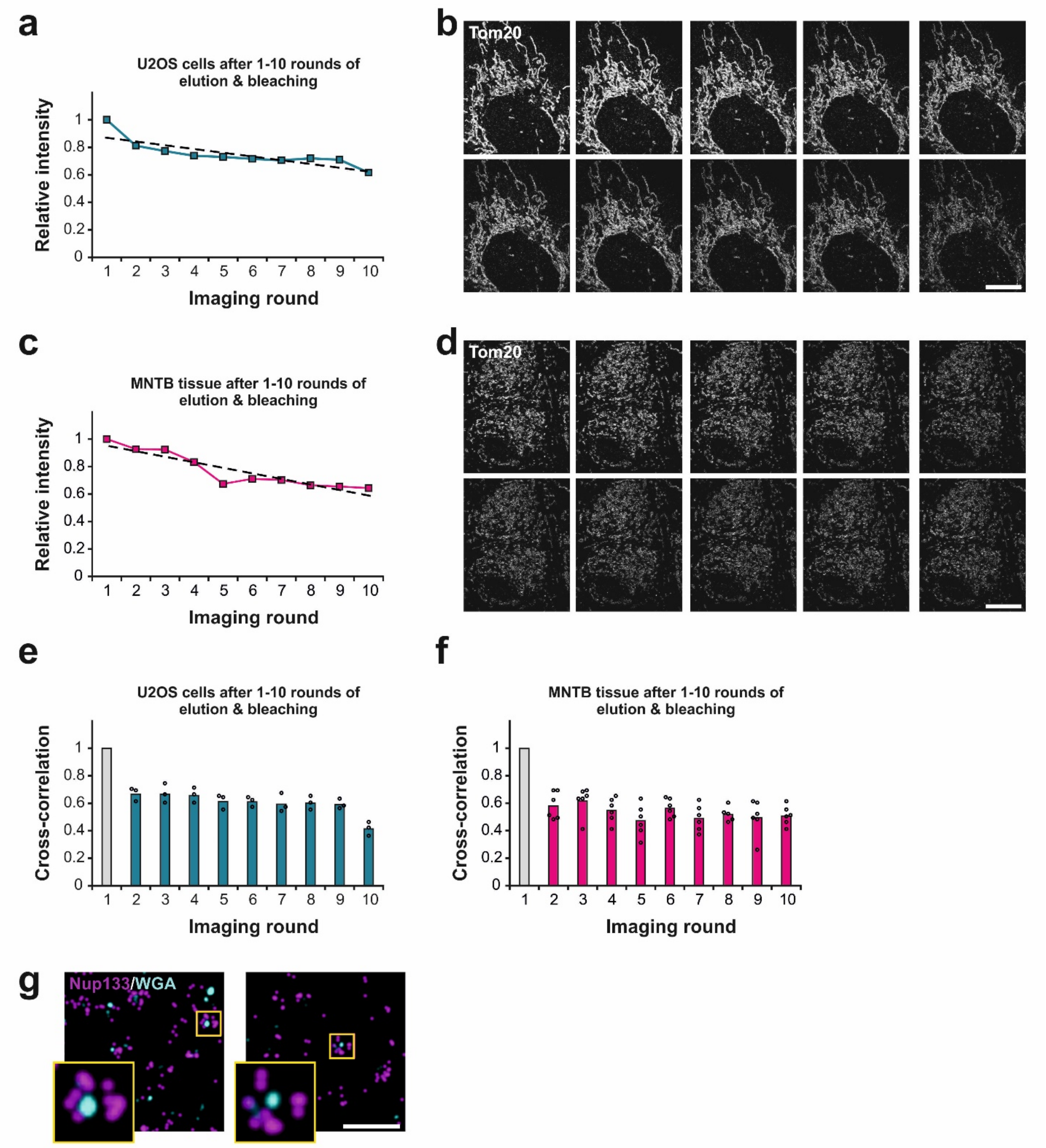

Supplementary Figure 9 | Signal preservation and structural integrity during repeated bleaching and elution. (**a**, **c**) Relative intensity and (**b**, **d**) representative images of Tom20 signal throughout 10 rounds of bleaching and elution (standard conditions; see Methods) demonstrate moderate signal loss as quantified by regression lines both in U2OS cells (a, b) and in MNTB tissue (c, d). (**e**, **f**) Structural integrity was evaluated using the cross-correlation method recently presented by Descloux et al. (2019)[2]. Super-resolution images from each round (1-10) were blurred with a sigma of 50 nm and compared to the image from the first round. The resulting cross-correlation coefficient can range between values of 0 (no correlation) and 1 (ideal correlation for identical images). The cross-correlation coefficient remained stable throughout imaging rounds 2-10; it ranged between 0.41 and 0.67 for U2OS cells (e) and 0.47 and 0.62 for tissue (f), suggesting a sustained structural integrity of

mitochondria. Note that comparing an image with itself as shown for round 1 results in a cross-correlation coefficient of 1 (open gray bars). (**g**) Nup133-Ypet expressing U2OS cells that underwent ten rounds of bleaching and elution were stained with anti-GFP nanobodies (also recognizing Ypet). Despite the very low epitope density (four epitopes per nuclear pore subunit), individual nuclear pore complexes are still apparent in the two exemplary panels and insets representing magnifications of boxed regions in imaging round 11. Data points (a, c) and bars (e, f) represent mean values. Scale bars correspond to 10 µm (b, d) and 500 nm (g). For sample numbers, see **Supplementary Table 5**. Note that in addition to representative images in **b** and **d** another 2 experiments were carried out, with similar designs and outcomes. Experiment shown in **g** has been carried out only once. Source data are provided as a Source Data file.

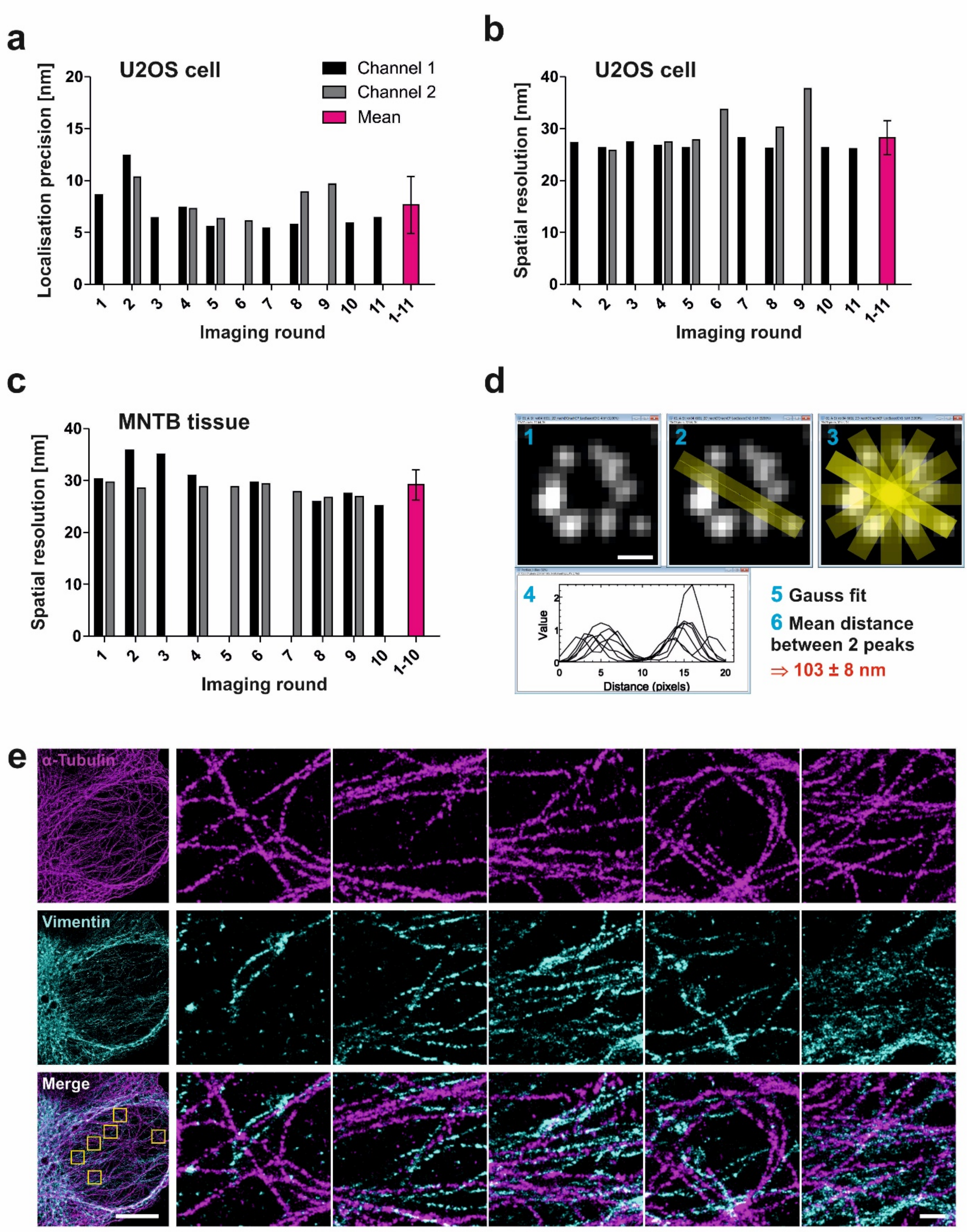

Supplementary Figure 10 | Localization precision and resolution of images acquired throughout the multiplex experiment. (**a**) Localization precision for the multiplex experiment in a U2OS cell shown in **Figure 2a** was estimated by quantifying the nearest neighbor (NeNa) distance between corresponding localizations from adjacent STORM frames[3]. NeNa analysis resulted in a mean NeNa distance of 7.5 ± 2 nm (mean ± SD) for all imaging rounds, indicating a very high localization precision. For this analysis we used the NeNa algorithm of LAMA software[4]. This was also confirmed by a decorrelation analysis[2] resulting in a high estimated

average spatial resolution of ~28 nm for the experiment in cells (**b**) shown in **Figure 2a** and ~29 nm for the experiment in tissue (**c**) shown in **Figure 3c**. (**d**) Analysis of the nuclear pore complex (NPC) metrics based on the Nup133 signal from the multiplex experiment in U2OS cells (**Fig 2a**) results in an average diameter of 103 ± 8 nm, which is within the range described in literature[5]. For this analysis, using a custom-written ImageJ script, the center of mass of an NPC was found (1) and a line selection with a width of 30 nm was laid through the NPC (2) and rotated around the center of mass in 30° steps resulting in six line selections (3). Six line plots were generated (4) per NPC, their peaks were Gauss-fitted (5) by a custom-written Matlab code and the mean distance between the two peaks was calculated and averaged among 10 NPCs tested (6). (**e**) Overview images (first column of panels) showing α-tubulin (magenta), vimentin (cyan) staining and the merged image. Representative magnifications (panel column 2-6) of the five yellow-boxed regions demonstrate that despite a rather low labeling efficiency individual microtubules and intermediate filaments can be discerned within the dense meshwork. (a-c) Magenta bars represent mean values among all analyzed image rounds ± SD. Scale bars correspond to 50 nm (d), 10 µm (e, panel column 1), and 500 nm (e, panel columns 2-6). For sample numbers, see **Supplementary Table 5**. Source data are provided as a Source Data file.

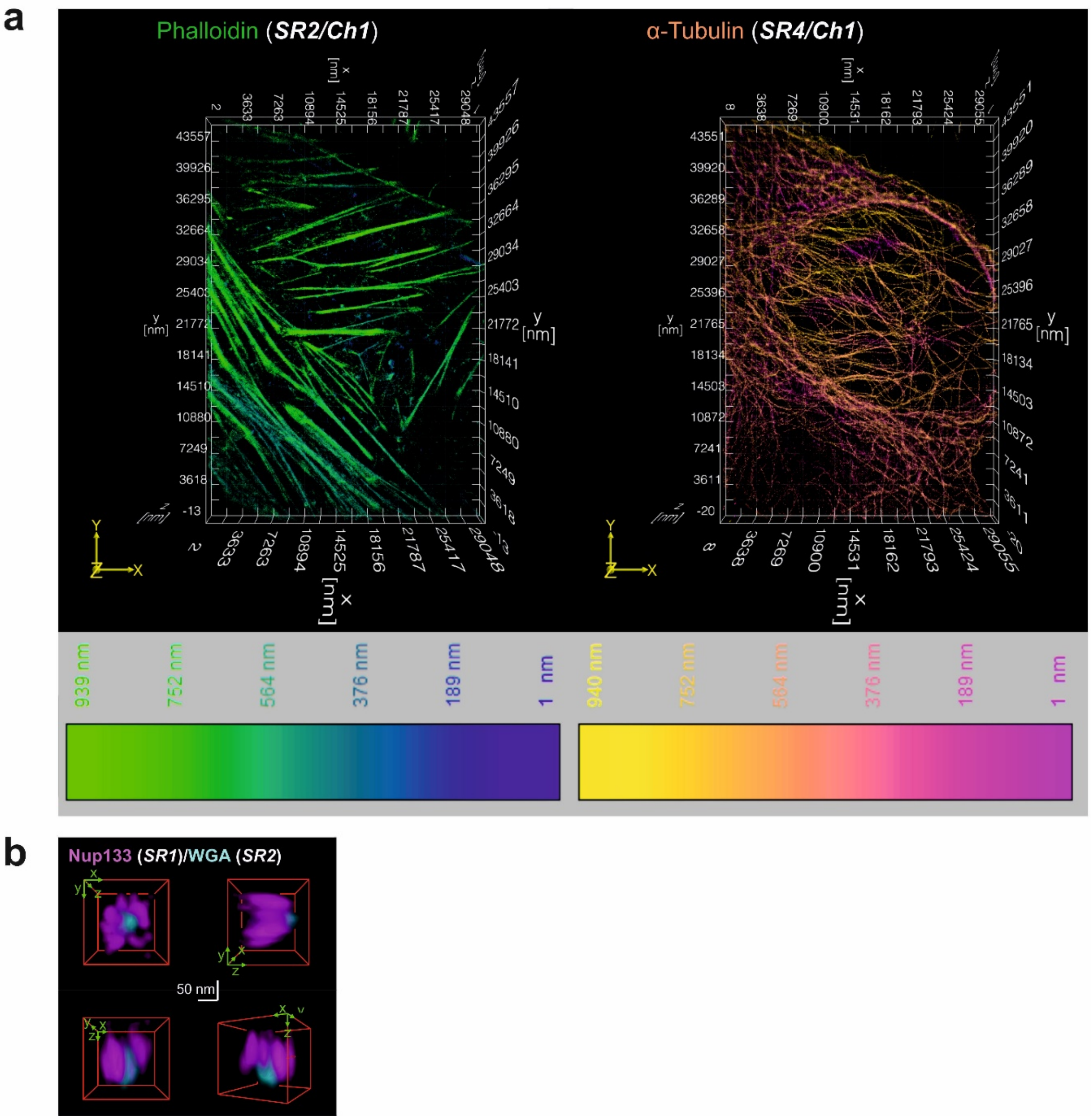

Supplementary Figure 11 | Examples showing 3D visualization of selected STORM images. (**a**) Three-dimensional representation of the phalloidin staining (left panel) and α-tubulin staining (right panel) from the multiplex experiment shown in **Figure 2a** using ViSP software[6]. (**b**) Second example (for first example, see **Figure 2d**) of a nuclear pore complex rendered in 3D, demonstrating high axial resolution and robust fiducial-based registration precision. ImageJ was used for 3D visualization in **b**.

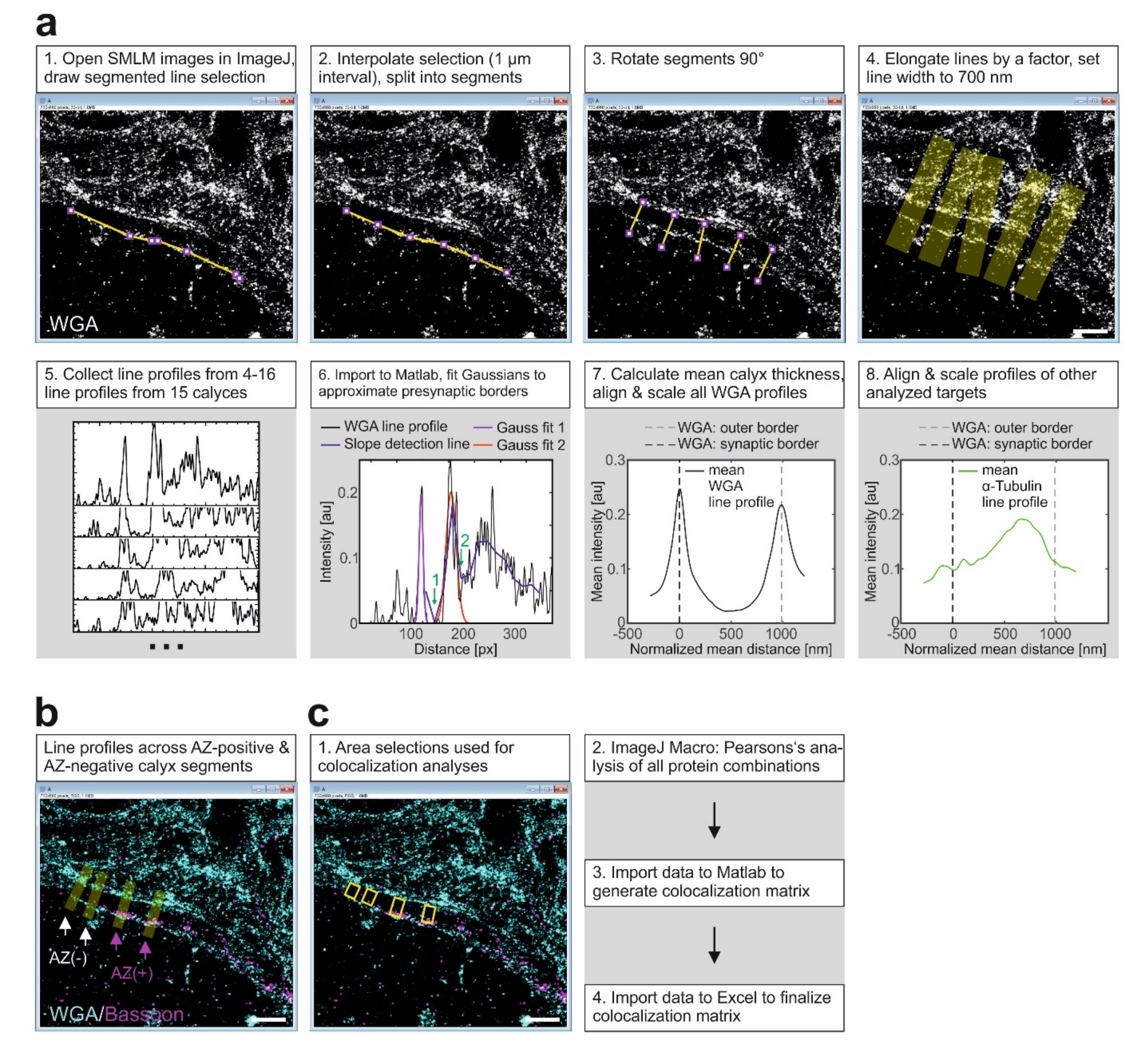

Supplementary Figure 12 | Workflow for analysis of presynaptic architecture of the calyx of Held. (**a**) The distribution of presynaptic proteins was analyzed using a custom ImageJ[7] plugin. (1) Manual outlines of the inner border of the calyx based on WGA stainings, aided by stainings of active zone (AZ) marker Bassoon and synaptic vesicle marker VGlut. (2) The line selection is automatically interpolated and split into segments. (3) Segments are rotated 90° to ensure perpendicular position of line profiles relative to the inner calyx membrane. (4) Lines are elongated to cover the whole presynapse and their width is set to a desired value (for this analysis to 700 nm). (5) The 'multi plot' command collects all line profiles in a text file for Matlab import. (6) Peaks corresponding to calyceal borders are fitted by Gaussian functions. For this, the inner calyx border is first localized within a defined window; this fit is very reliable as the distance between the line profile origin and the inner border is nearly constant. As different calyces and calyx stretches have variable thickness, the second (outer) border is identified by two intensity minima: a first one that directly follows the first border, and a subsequent one (green arrows in panel 6). The strong intensity peak in between those minima is identified as the second border, and its position approximated by a Gaussian function. (7) Mean calyx thickness is determined from aligned and scaled profiles. (8) All profiles of WGA and proteins of interest are averaged, scaled, and aligned according to the mean calyx thickness. (**b**) For the analysis of AZ-specific protein distribution, two line profiles in AZ-negative (AZ(-)) and two

profiles in AZ-positive (AZ(+)) presynaptic regions were drawn per calyx. (**c**) For colocalization analysis, line profiles were truncated to specifically cover only the presynaptic area and converted to area selections (1). Using a custom ImageJ plugin, all possible combinations of targets underwent Pearson's colocalization analysis (2). All data were imported to Matlab to generate colocalization matrices (3), which were imported to Excel where they were averaged and finalized. (**a**-**c**) Scale bars correspond to 1 µm.

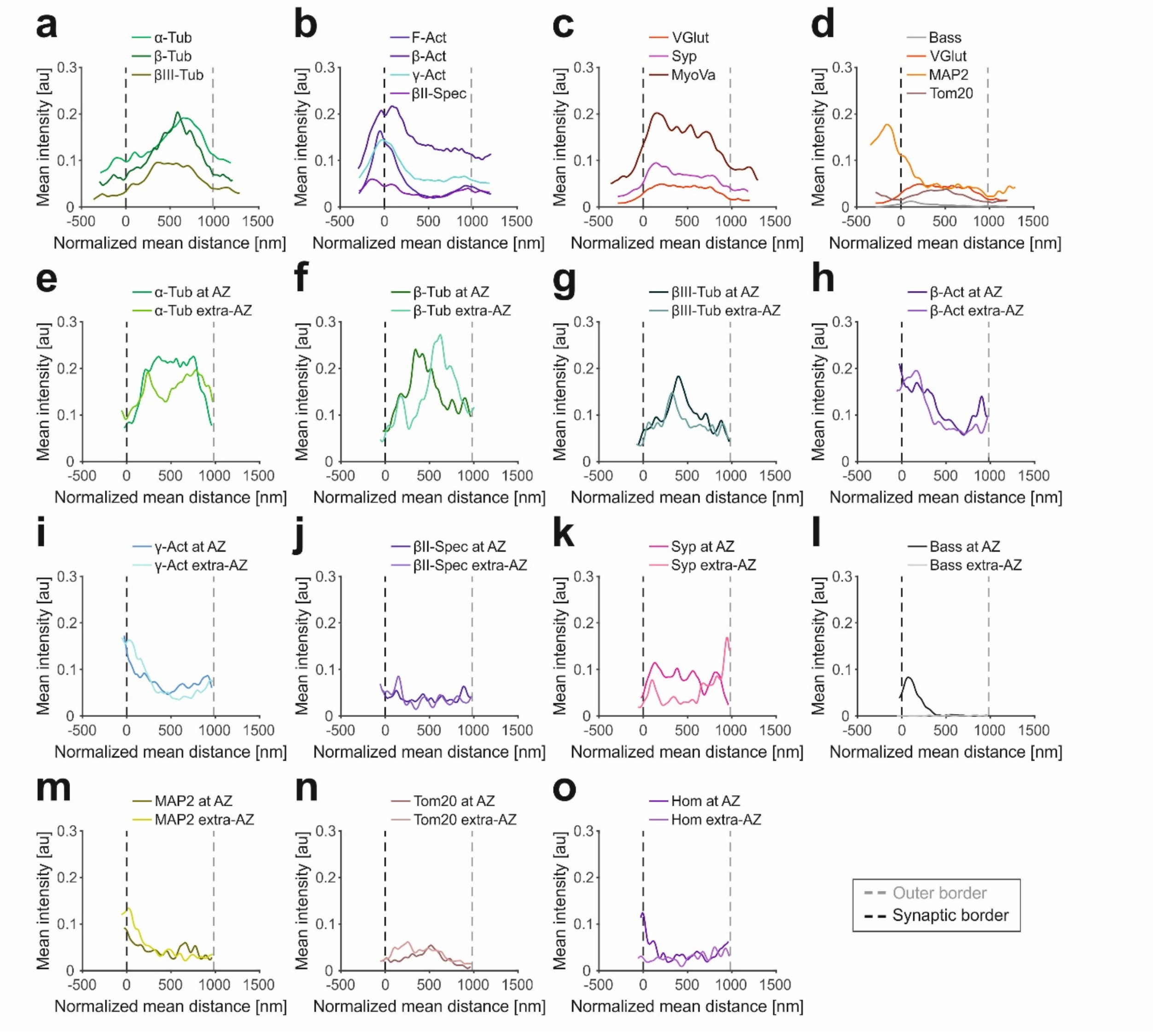

Supplementary Figure 13 | Averaged line profiles of global and active zone-specific protein distributions. (**a**) All analyzed tubulin (Tub) family members show a similar distribution. (**b**) Labels against polymerized actin (Act) or against α- and β-actin isoforms show similar profiles. Interestingly, βII-spectrin (βII-Spec) distribution resembles that of actin. (**c**) Motor protein myosin Va (MyoVa) is distributed throughout the presynapse in line with the distribution of its cargo reflected by synaptic vesicle markers VGlut and synaptophysin 1 (Syp). (**d**) Different markers reflecting the overall geometry of the calyx of Held. (**e-o**) Distribution of proteins in active zone (AZ) positive versus AZ-negative calyx regions. For sample numbers, see **Supplementary Table 5**.

		VGlut 1	Syp 2	MyoVa 3	Bass 4	F-Act 5	β-Act 6	γ-Act 7	βII-Spec 8	WGA 9	α-Tub 10	β-Tub 11	βIII-Tub 12	MAP2 13	Tom20 14
VGlut	1		0.06	0.11	0.03	0.01	-0.01	0.03	-0.01	-0.02	-0.02	0.02	0.02	0.06	0.02
Syp	2	0.09		-0.03	-0.01	0.01	0.01	0.00	-0.01	0.01	0.04	0.01	0.01	0.01	0.07
MyoVa	3	0.13	0.02		0.00	0.03	0.05	0.06	0.01	-0.06	0.04	0.09	0.05	0.07	0.02
Bass	4	0.03	0.01	0.06		0.07	0.03	0.04	-0.02	0.10	-0.05	-0.03	-0.02	0.02	0.00
F-Act	5	0.00	-0.05	-0.01	0.00		0.10	0.09	0.01	0.09	-0.05	-0.03	-0.07	0.03	-0.02
β-Act	6	0.00	0.02	-0.03	0.01	0.15		0.14	0.06	0.06	0.04	-0.04	-0.05	0.03	0.00
γ-Act	7	0.04	0.04	0.03	-0.01	0.19	0.13		0.07	0.08	0.06	0.00	0.02	0.06	-0.01
βII-Spec	8	0.00	0.02	0.04	-0.02	0.07	0.04	0.01		0.02	0.03	0.06	0.06	0.03	0.00
WGA	9	-0.01	-0.04	-0.06	-0.03	0.19	0.01	0.07	0.01		-0.09	-0.11	-0.05	0.01	-0.02
α-Tub	10	0.04	0.07	0.05	-0.01	-0.01	0.06	0.08	0.06	-0.06		0.21	0.19	0.03	0.02
β-Tub	11	0.02	0.04	0.04	-0.01	-0.05	0.02	0.01	0.06	-0.04	0.24		0.19	0.00	0.06
βIII-Tub	12	0.02	0.00	0.05	0.01	-0.03	-0.01	0.00	0.00	-0.06	0.21	0.16		0.05	0.05
MAP2	13	0.07	0.00	0.05	0.00	0.06	0.01	0.02	-0.01	0.03	-0.01	-0.04	-0.01		0.01
Tom20	14	-0.02	0.04	0.02	0.00	-0.02	0.00	0.01	-0.02	-0.05	0.08	0.00	0.00	0.01	

Active zone area

Active zone-free area

-0.15
-0.10
-0.05
0.00
0.05
0.10
0.15
0.20
0.25

Supplementary Figure 14 | Colocalization matrix. Pearson's r values for the colocalization matrix shown in **Figure 3m**.

Supplementary Note 1

*Extended information relating to control experiments assessing signal removal after each staining round (**Supplementary Figure 6a**)*

We thoroughly assessed the performance of re-staining, bleaching, and elution using Tom20 staining as a test case. For each control condition (**Supplementary Figure 6a**), three sequential *d*STORM imaging experiments (with three acquisitions per experiment and three ROIs per acquisition) were conducted, from which we quantified the fluorescence intensity by determining the number of single-molecule localizations (**Supplementary Figure 6b**).

First, we determined the fluorescence intensity in three consecutive *d*STORM experiments performed with identical imaging conditions (data set #1 in **Supplementary Figure 6a**). The number of localizations of the second and third experiments were normalized with respect to the first experiment. As expected, *d*STORM imaging decreased fluorescence intensity in subsequent imaging experiments by photobleaching some of the fluorophore labels (see Supplementary Figure 6c for graphical illustration).

Second, we performed an experiment with three consecutive *d*STORM acquisitions, but labeled again with the same fluorophore-labeled secondary antibody prior to the third imaging round (data set #2). We found similar fluorescence intensities as in the first data set, despite re-labeling with the secondary antibody. This result tells us that the secondary antibody labeling reached saturation.

Third, we recorded three *d*STORM data sets, yet now photobleached the remaining fluorescence signal after the first acquisition by applying high irradiation intensities (data set #3, see Methods). The second imaging round showed us that bleaching was efficient since the fluorescence intensity dropped to background level. Prior to the third experiment, we relabeled the sample again with the same fluorophore-labeled secondary antibody. We found a recovery of the fluorescence intensity, which we explain by photo-induced unbinding of the secondary antibody through high-intensity illumination of the sample during the photobleaching step. We find similar ratios of recovery (about 45%) as reported in the literature[6].

Fourth, we explored the efficiency of elution (data set #4, see Methods). After the first imaging round, we eluted the antibodies, and we observed a decrease in fluorescence intensity. A third imaging round shows a decreased intensity as observed in condition one and two.

Fifth, we explored the efficiency of both photobleaching and elution after the first imaging round (data set #5). After the initial staining, we first exposed the sample to the elution buffer followed by a photobleaching step. We found the fluorescence intensity to drop to background level. Re- staining with the same secondary antibody recovers fluorescence signal (to 8.6%), which we attribute to photo-unbinding of the secondary antibody[8].

Sixth, we show that the combination of photobleaching and elution is highly efficient, if another primary antibody (other than Tom20; in this case Fibrillarin) competing for the same secondary antibody is introduced before the last acquisition round (data set #6). This would represent the typcial workflow of a multiplex experiment.

In summary, for optimal performance of our approach, we recommend to design a re-labeling experiment such that (i) both elution and photobleaching steps are integrated in the workflow, and that (ii) the labeling sequence is designed such that the species of secondary antibodies changes every imaging round. See **Supplementary Note 2** for a detailed description of experimental design.

Supplementary Note 2

Set of rules helping the user to design an optimal multiplex experiment

Here, we list some main rules that help to design an optimal multiplex experiment. Depending on the specific scientific question, the experimenter can reorder the rules by prioritizing rules that help to get optimal results for particular structures of interest.

- Stain targets with low epitope density in early staining rounds.

- Use labels with low labelling efficiency in early staining rounds.

- Use high affinity antibodies in late staining rounds.

- Try to avoid elution-based label removal for as many rounds as possible. Bleaching is sufficient as long as not interfering non-antibody labels and antibodies from different species are used.

- Avoid primary antibodies from same species in consecutive rounds.

- For direct labels (such as lectins) that cannot interfere with other labels use bleaching only.
- For primary/secondary antibody labeling use elution only.

- Do not stain targets with high number of epitopes or use labels with high labeling efficiency together with targets with low epitope numbers or low labeling capacity in one round. This is because cross-talk originating from demixing can lower the signal to background ratio for the low- signal target.

- Cytoskeleton components are more sensitive to sample treatment and should therefore be labeled in early staining rounds if very detailed ultrastructural data is required.

- If using primary antibodies from same species in consecutive rounds, alternate secondary antibody labels (Alexa 647 and CF680). This allows to distinguish between cross-talk from fluorophores of remaining secondary antibodies or from remaining primary antibodies.

- If imaging in PAINT (points accumulation for imaging in nanoscale topography) mode, image fluorescent fiducials before application of PAINT dyes. Otherwise fiducial detection might be impeded due to the high background produced by PAINT dyes in solution.

- The best possible correlation of two targets can be achieved by imaging in the same round (channel 1 and 2).

Supplementary Note 3

Supplementary discussion of registration precision between different staining rounds for cells or tissues

The serial re-staining approach used here requires a high-fidelity registration of images from each staining round to reliably derive conclusions on the positioning of each protein with regard to all other proteins investigated. We achieved this by imaging fiducial beads positioned between the sample and the coverslip for every staining round. Hence, assuming an invariant position of each bead, the registration procedure should work with similar precision for images acquired in any staining round. Protein positions determined from the first staining round should be equally comparable to those of the second or 10th staining round. However, if the position of the fiducial beads varies to a small extent during the long duration of a typical multiplexed imaging experiment, deviations will occur, in particular for protein positions determined from imaging rounds far apart, but not much affecting neighboring staining rounds. This relationship is quite evident in **Figures 2b** and **3d.** Noticeably, the registration precision was overall better for super-resolution images acquired in tissue (**Figure 3d**) compared to cells (**Figure 2b**). We attribute this to a differential stability of the fiducials in these two imaging situations as detailed in the following. In contrast to the flat 400 nm sections, cultured U2OS cells have a substantial thickness of several micrometers. Due to their thickness and other mechanical properties, cells may not homogeneously adhere to the glass surface, resulting in a slight partial detachment with each additional staining round. This could in turn loosen fiducial beads stuck underneath the cells. Slight changes in cell volume with increasing staining and elution rounds may cause a similar effect. Therefore, to re-establish a focal plane in late imaging rounds comparable to that from the initial round, the focus would have to be placed more and more deeply into the sample, resulting in less focused fiducial beads, and thus, less precise fitting and a higher registration error. Indeed, we once had to manually readjust the focus in round five of the multiplex experiment in cells (**Figure 2a**). In the much more homogeneous tissue sections, these effects will impact registration precision much less, consistent with the experimental data (**Figures 2b** and **3d**). Hence, we have focused our quantitative analyses on neuronal tissue samples to minimize these complications. Moreover, for the analysis of protein relationships we performed five multiplex experiments with target proteins imaged in a different order. Thereby, we balanced out possible effects arising from misalignment in late compared to early imaging rounds. Future work may include the use of fiducials covalently fixed to the cover slip using nano-engineering procedures.

Supplementary references

1. Edelstein, A. D. *et al.* Advanced methods of microscope control using µManager software. *J. Biol. Methods* **1**, 10 (2014).
2. Descloux, A., Grußmayer, K. S. & Radenovic, A. Parameter-free image resolution estimation based on decorrelation analysis. *Nat. Methods* **16**, 918–924 (2019).
3. Endesfelder, U., Malkusch, S., Fricke, F. & Heilemann, M. A simple method to estimate the average localization precision of a single-molecule localization microscopy experiment. *Histochem. Cell Biol.* **141**, 629–638 (2014).
4. Malkusch, S. & Heilemann, M. Extracting quantitative information from single-molecule super-resolution imaging data with LAMA – LocAlization Microscopy Analyzer. *Sci. Rep.* **6**, 34486 (2016).
5. Szymborska, A. *et al.* Nuclear pore scaffold structure analyzed by super-resolution microscopy and particle averaging. *Science (80-.).* (2013). doi:10.1126/science.1240672
6. Beheiry, M. El & Dahan, M. ViSP: representing single-particle localizations in three dimensions. *Nat. Methods* **10**, 689–690 (2013).
7. Schindelin, J. *et al.* Fiji: an open-source platform for biological-image analysis. *Nat. Methods* **9**, 676–682 (2012).
8. Heinze, K. G., Costantino, S., De Koninck, P. & Wiseman, P. W. Beyond Photobleaching, Laser Illumination Unbinds Fluorescent Proteins. *J. Phys. Chem. B* **113**, 5225–5233 (2009).

Supplementary Table 1: Experimental workflow for multiplex experiment in U2OS cells shown in **Figure 2a**

SR	Treatment	Agent	Condition	Duration
1	staining	GFP nanobodies AF647 (1:80)	in 0.5% FCS	1 h
	STORM	MEA buffer	20.000 frames at 30 ms rate	~11 min
	bleaching	in PBS	100% 661 nm, 50% 405 laser	4 min
2	staining	Phalloidin-AF647 (1:30), WGA-CF680 (1:500)	in PBS	20 min
	STORM	MEA buffer	20.000 frames at 30 ms rate	~11 min
	bleaching	in PBS	100% 661 nm, 50% 405 laser	4 min
3	staining	CHC17-AF647 antibody (1:100)	in 0.5% FCS	1 h
	STORM	MEA buffer	20.000 frames at 30 ms rate	~11 min
	bleaching	in PBS	100% 661 nm, 50% 405 laser	4 min
4	staining 1. AB	α-Tubulin, ms (1:100) & Paxillin, rb (1:300)	in 0.5% FCS	1 h
	staining 2. AB	ms-AF647 (1:500) & rb-CF680 (1:500)	in 0.5% FCS	1 h
	STORM	MEA buffer	20.000 frames at 30 ms rate	~11 min
	elution	0.1% SDS, pH 13		15 min
	bleaching	in PBS	100% 661 nm, 50% 405 laser	4 min
5	staining 1. AB	Vimentin, ch (1:500) & GM130, ms (1:500)	in 0.5% FCS	1 h
	staining 2. AB	ch-AF647 (1:500) & ms-CF680 (1:500)	in 0.5% FCS	1 h
	STORM	MEA buffer	20.000 frames at 30 ms rate	~11 min
	elution	0.1% SDS, pH 13		15 min
	bleaching	in PBS	100% 661 nm, 50% 405 laser	4 min
6	staining 1. AB	Fibrillarin, rb (1:200)	in 0.5% FCS	1 h
	staining 2. AB	rb-CF680 (1:500)	in 0.5% FCS	1 h
	STORM	MEA buffer	20.000 frames at 30 ms rate	~11 min
	elution	0.1% SDS, pH 13		15 min
	bleaching	in PBS	100% 661 nm, 50% 405 laser	4 min
7	staining 1. AB	Tom20, rb (1:80)	in 0.5% FCS	1 h
	staining 2. AB	rb-AF647 (1:500)	in 0.5% FCS	1 h
	STORM	MEA buffer	20.000 frames at 30 ms rate	~11 min
	elution	0.1% SDS, pH 13		15 min
	bleaching	in PBS	100% 661 nm, 50% 405 laser	4 min
8	staining 1. AB	Lamin A/C, ch (1:500) & PDI, ms (1:1000)	in 0.5% FCS	1 h
	staining 2. AB	ch-AF647 (1:500) & ms-CF680 (1:500)	in 0.5% FCS	1 h
	STORM	MEA buffer	20.000 frames at 30 ms rate	~11 min
	elution	0.1% SDS, pH 13		15 min
	bleaching	in PBS	100% 661 nm, 50% 405 laser	4 min
9	staining 1. AB	Rab5, rb (1:200)	in 0.5% FCS	1 h
	staining 2. AB	rb-CF680 (1:500)	in 0.5% FCS	1 h
	STORM	MEA buffer	20.000 frames at 30 ms rate	~11 min
	elution	0.1% SDS, pH 13		15 min
	bleaching	in PBS	100% 661 nm, 50% 405 laser	4 min
10	staining 1. AB	EEA1, rb (1:1000)	in 0.5% FCS	1 h
	staining 2. AB	rb-AF647 (1:500)	in 0.5% FCS	1 h
	STORM	MEA buffer	20.000 frames at 30 ms rate	~11 min
	elution	0.1% SDS, pH 13		15 min
	bleaching	in PBS	100% 661 nm, 50% 405 laser	4 min
11	PAINT	MEA buffer containing 2 nM JF_{646}-Hoechst	20.000 frames at 50 ms rate	~17 min

SR = staining round; ms = mouse; ch = chicken; rb = rabbit; gp = guinea pig; 1. AB = primary antibody; 2. AB = secondary antibody

Supplementary Table 2: Experimental workflow for the multiplex experiment in the medial nucleus of the shown in **Figure 3c**

SR	Treatment	Agent	Condition	Duration
1	staining 1. AB	Bassoon (1:400), Homer 1/2/3 (1:500)	in 0.5% FCS	1 h
	staining 2. AB	ms-AF647 (1:500) & rb-CF680 (1:500)	in 0.5% FCS	1 h
	STORM	MEA buffer	20.000 frames at 30 ms rate	~11 min
	bleaching	in PBS	100% 661 nm, 50% 405 laser	4 min
2	staining	Phalloidin-AF647 (1:30), WGA-CF680 (1:500)	in PBS	20 min
	STORM	MEA buffer	20.000 frames at 30 ms rate	~11 min
	bleaching	in PBS	100% 661 nm, 50% 405 laser	4 min
3	staining 1. AB	CHC17-AF647 antibody (1:100)	in 0.5% FCS	1 h
	STORM	MEA buffer	20.000 frames at 30 ms rate	~11 min
	bleaching	in PBS	100% 661 nm, 50% 405 laser	4 min
4	staining 1. AB	MAP2, ch (1:500) & VGlut, gp (1:300)	in 0.5% FCS	1 h
	staining 2. AB	ch-AF647 (1:500) & gp-CF680 (1:500)	in 0.5% FCS	1 h
	STORM	MEA buffer	20.000 frames at 30 ms rate	~11 min
	elution	0.1% SDS, pH 13		15 min
	bleaching	in PBS	100% 661 nm, 50% 405 laser	4 min
5	staining 1. AB	GM130, ms (1:200)	in 0.5% FCS	1 h
	staining 2. AB	ms-CF680 (1:500)	in 0.5% FCS	1 h
	STORM	MEA buffer	20.000 frames at 30 ms rate	~11 min
	elution	0.1% SDS, pH 13		15 min
	bleaching	in PBS	100% 661 nm, 50% 405 laser	4 min
6	staining 1. AB	Rab3a, ms (1:250) & Tom20, rb (1:70)	in 0.5% FCS	1 h
	staining 2. AB	ms-AF647 (1:500) & rb-CF680 (1:500)	in 0.5% FCS	1 h
	STORM	MEA buffer	20.000 frames at 30 ms rate	~11 min
	elution	0.1% SDS, pH 13		15 min
	bleaching	in PBS	100% 661 nm, 50% 405 laser	4 min
7	staining 1. AB	α-Tubulin, ms (1:500)	in 0.5% FCS	1 h
	staining 2. AB	ms-CF680 (1:500)	in 0.5% FCS	1 h
	STORM	MEA buffer	20.000 frames at 30 ms rate	~11 min
	elution	0.1% SDS, pH 13		15 min
	bleaching	in PBS	100% 661 nm, 50% 405 laser	4 min
8	staining 1. AB	γ-Actin, ms (1:50) & Synaptophysin1, rb (1:100)	in 0.5% FCS	1 h
	staining 2. AB	ms-AF647 (1:500) & rb-CF680 (1:500)	in 0.5% FCS	1 h
	STORM	MEA buffer	20.000 frames at 30 ms rate	~11 min
	elution	0.1% SDS, pH 13		15 min
	bleaching	in PBS	100% 661 nm, 50% 405 laser	4 min
9	staining 1. AB	α/β-Synuclein, rb (1:200) & PDI, ms (1:500)	in 0.5% FCS	1 h
	staining 2. AB	rb-AF647 (1:500) & ms-CF680 (1:500)	in 0.5% FCS	1 h
	STORM	MEA buffer	20.000 frames at 30 ms rate	~11 min
	elution	0.1% SDS, pH 13		15 min
	bleaching	in PBS	100% 661 nm, 50% 405 laser	4 min
10	PAINT	MEA buffer containing 2 nM JF_{646}-Hoechst	20.000 frames at 50 ms rate	~17 min

SR = staining round; ms = mouse; ch = chicken; rb = rabbit; gp = guinea pig; 1. AB = primary antibody; 2. AB = secondary antibody

Supplementary Table 3: All antibodies and other labels used in this work

Antigen/target	Type of label	Conjuga-ted fluo-rophore	Host species	Clonality (clone №)	Relevant species reactivity*	Company	ID/order number	Lot number	Appli-cation
CHC17	1. AB (IgG1)	AF647	ms	monocl. (X22)	hu, rt	Novus	NB300-613AF647	SL258005-082318	1:40
DNA	bisbinzimidine (Hoechst)	JF646	n.a.	n.a.	n.a.	- (Luke Lavis lab)	-	-	2 nM
F-Actin	toxin (Phalloidin)	AF647	n.a.	n.a.	n.a.	Thermo Fisher	A22287	1750839	1:30
GFP, CFP, and YFP variants (i.a. Ypet),	nanobody	AF647	produced in *E. coli*	biclonal (1H1-1B2)	n.a.	NanoTag	customized conjugation	-	1:80
N-acetylglucos-aminyl, sialic acid	lectin (WGA)	CF680	-	-	-	Biotium	29029	16W0329	1:500
β-Actin	1. AB (IgG1)	-	ms	monocl. (AC-15)	hu	Abcam	AB6276	GR66278-17	1:50
γ-Actin	1. AB (IgG1)	-	ms	monocl. (2-2)	hu	Santa Cruz	sc-65636	G2309	1:50
Bassoon	1. AB (IgG2)	-	ms	monocl.	rt	Enzo Life Sciences	SAP7F407	#04011024	1:400
EEA1	1. AB (IgG)	-	rb	polycl.	hu	Abcam	AB2900	-	1:1000
Fibrillarin	1. AB (IgG)	-	rb	polycl.	hu	Abcam	AB5821	135416/1-4	1:200
GM130	1. AB (IgG1)	-	ms	monocl. (35)	hu, rt	BD	610822	6217559	1:500
Homer 1/2/3	1. AB (IgG)	-	rb	polycl.	rt	SYSY	160103	160103/5	1:500
Lamin A/C	1. AB (IgY)	-	ch	polycl.	hu	VWR	BSENC-1698-100	C-1698-300	1:1000
MAP2	1. AB (IgY)	-	ch	polycl.	rt	SYSY	188006	188006/1	1:500
Myosin Va	1. AB (IgG)	-	rb	polycl.	rt	Merck	M4812	127K4812	1:100
Paxillin	1. AB (IgG)	-	rb	monocl. (Y113)	hu	Abcam	AB32084	GR215958-32	1:300
PDI	1. AB (IgG1)	-	ms	monocl. (1D3)	hu, rt	Enzo Life Sciences	ADI-SPA-891	5061344	1:1000
Rab3a	1. AB (IgG1)	-	ms	monocl. (42.2)	rt	SYSY	107111	-	1:250
Rab5	1. AB (IgG)	-	rb	polycl.	rt	Abcam	AB18211	793878	1:200
βII-Spectrin	1. AB (IgG1)	-	ms	polycl.	rt	BD	612562	3028739	1:100
Synaptophysin 1	1. AB (IgG)	-	rb	polycl.	rt	SYSY	101102	101002/26	1:100
α/β-Synuclein	1. AB (IgG)	-	rb	polycl.	rt	SYSY	128003	128003/1	1:300
Tom20	1. AB (IgG)	-	rb	polycl.	hu, rt	Santa Cruz	sc-11415	D0615	1:80
α-Tubulin	1. AB (IgG1)	-	ms	monocl. (DM1A)	hu, rt	Sigma-Aldrich	T6199	115M4796V	1:500
β-Tubulin	1. AB (IgG)	-	rb	polycl.	rt	Dianova	DLN-15304	249P1209F1	1:200
βIII-Tubulin	1. AB (IgG1)	-	ms	monocl. (TU-20)	rt	Abcam	AB7751	GR59358-7	1:400
VGlut 1	1. AB (IgG)	-	gp	polycl.	rt	Merck	AB5905	2647529	1:200
Vimentin	1. AB (IgY)	-	ch	polycl.	hu	Merck	AB5733	2262642	1:500
ch IgY (H+L)	2. AB (IgG)	AF647	gt	polycl.	ch	Thermo Fisher	A21449	1806124	1:500
gp IgG (H+L)	2. AB (IgG)	CF680	rb	polycl.	gp	Merck	SAB4600204	12C01404	1:500
ms IgG (H+L)	2. AB (IgG)	AF647	gt	polycl.	ms	Thermo Fisher	A21235	1922319	1:500
ms IgG (H+L)	2. AB (IgG)	CF680	gt	polycl.	ms	Merck	SAB4600199	13C0903	1:500
rb IgG (H+L)	2. AB (IgG)	AF647	gt	polycl.	rb	Thermo Fisher	A21245	1981173	1:500
rb IgG (H+L)	2. AB (F(ab')2)	CF680	gt	polycl.	rb	Merck	SAB4600362	11C0316	1:500

WGA = wheat germ agglutinin; 1. AB = primary antibody; 2. AB = secondary antibody; AF647 = Alexa Fluor 647; ms = mouse; rb = rabbit; gp = guinea pig; gt = goat; hu = human; rt = rat; n.a. = not applicable; *stated by vendor

Supplementary Table 4: maS[3]TORM components

Devices/components	Company	Order/model/serial №	№[1]
**Objective-related**			
Objective: 100x, 1.49 Na, 0.1 mm working distance	Olympus	UAPON 100XOTIRF	#9
Stage: xy, ca. 123 & 103 mm travel distance	SmarAct	custom-designed[2]	n.i.
Controller for xy-stage & piezo mirror positioner	SmarAct	HCU-3DM-USB-TAB	n.i.
**Focus lock**			
Laser diode: 785 nm, 5 mW	Thorlabs	L785P5	#16
Dichroic mirror T770 dcspxrxt	Chroma	T770dcspxrxt	#17
Controller & power supply for 785 nm diode	-/custom-made	-/custom-made	n.i.
Piezo mirror positioner: 123 µm range	SmarAct	SLC-24180-M-E	#18
Quadrant silicon photodiode: red enhanced	Laser Components	SD 197-23-21-041	#19
Piezo objective positioner: 100 µm range	Physik Instrumente	P-726.1CD	#20
Controller for piezo objective positioner	Physik Instrumente	E-709.CRG	n.i.
Board processing output from quadrant diode, feeding piezo objective positioner	Laser Components	LC-301 DQD	n.i.
Dichroic mirror ZT405/488/561/647rpc-UF2	Chroma	IN040787	#8
**Excitation pathway**			
Laser: 405 nm, Cube, 100 mW	Coherent	1170506	#1
Laser: 488 nm, Obis, 100 mW	Coherent	1226420	#2
Laser: 561 nm, Obis, 100 mW	Coherent	1253302	#3
Laser: 661 nm, Cube, 100 mW	Coherent	141667501	#4
Clean-up filter for 661 nm laser: ZET 660/20	AHF	F49-660	n.i.
Beam splitter 525 DCXRU	AHF	F33-526	#5
Beam splitter ZT442rdc	AHF	F48-442	#6
Beam splitter 590 DCXR	Chroma	NC316551	#7
Lense mounts (2x) for 30 mm rail system	Thorlabs	CXY1	n.i.
Lense: focal length 10 mm	Thorlabs	AC080-010-A	n.i.
Lense: focal length 100 mm	Thorlabs	AC254-100-A	n.i.
**Emission pathway**			
Tube lense: focal length 180 mm	Thorlabs	AC508-180-A	n.i.
Cylindrical lense for 3D imaging: 1000 mm focal length	Thorlabs	LJ1516RM-A	#10
Notch filter NF658-26 for 660 nm excitation	Thorlabs	NF658-26	n.i.
Slit to narrow channels projected to camera chip: 3.4 mm width	-/custom-made	-/custom-made	#11
Lense: focal length 250 mm	Thorlabs	AC508-250B	n.i.
Motorized filter wheel for 6 filters	Thorlabs	FW102C	#12
Emission filter 525/45 BrightLine HC: for 488 nm laser	AHF	F37-521	n.i.
Emission filter 605/50 ET Bandpass: for 561 nm laser	AHF	F49-605	n.i.
Emission filter 700/75 ET Bandpass: for 661 nm laser	AHF	F47-700	n.i.
Dichroic mirror 690 DCXR	AHF	F33-692	#13
Lenses (2x): focal length 300 mm	Thorlabs	AC508-300B	n.i.
Prism: right angle, 40 mm	Thorlabs	PS912	#14
Camera: iXon Ultra 897	Andor	DU-897U-CS0-#BV	#15
**Pipetting robot**			
Robotic xyz-autosampler: PAL3 RTC	Axel Semrau	AS-CC-7201	n.i.
Liquid tool: CTC for 1000 µl syringes	Axel Semrau	AS-CC-7501	n.i.
Syringe: 1000µl, CTC C-Line	Axel Semrau	AS-CC-7627	n.i.
Liquid tool: CTC for 100 µl syringes	Axel Semrau	AS-CC-7500	n.i.
Syringe: 100µl, CTC X-Line	Axel Semrau	AS-CC-7605	n.i.
Washing station for PAL3	Axel Semrau	AS-CC-7562	n.i.
Tray holder	Axel Semrau	AS-CC-7400	n.i.
Trays for 54 vials (2 ml)	Axel Semrau	AS-CC-7406	n.i.

[1]Number in Supplementary Figure 1a or not indexed (n.i.); [2]design by Jonas Ries

Supplementary Table 5: Exact number of experiments, samples, and selections for all quantified analyses

Analysis and data subset	№ of experiments	№ of cells per experiment/ total № of cells	№ of line profiles or ROIs per cell in each experiment (*E*)
**Line profile analysis of the overall protein distribution at the calyx of Held (Fig. 3f, g; Fig. S13a-d)**			
WGA, α-Tubulin, F-Actin, γ-Actin, VGlut 1, Bassoon, Tom20	5	3/15 calyces	***E1:*** 5, 16, 16; ***E2:*** 8, 7, 8; ***E3:*** 13, 9, 10; ***E4:*** 10, 13, 13; ***E5:*** 15, 4, 15
β-Tubulin	3	3/9 calyces	***E2:*** 8, 7, 8; ***E3:*** 13, 9, 10; ***E4:*** 10, 13, 13
βIII-Tubulin	3	3/9 calyces	***E2:*** 8, 7, 8; ***E3:*** 13, 9, 10; ***E5:*** 15, 4, 15
β-Actin	3	3/9 calyces	**E3:** 13, 9, 10; **E4:** 10, 13, 13; **E5:** 15, 4, 15
Homer 1/2/3, Synaptophysin 1	4	3/12 calyces	***E1:*** 5, 16, 16; ***E3:*** 13, 9, 10; ***E4:*** 10, 13, 13; ***E5:*** 15, 4, 15
MAP2	3	3/9 calyces	***E1:*** 5, 16, 16; ***E2:*** 8, 7, 8; ***E3:*** 13, 9, 10
Myosin Va, βII-Spectrin	4	3/12 calyces	***E2:*** 8, 7, 8; ***E3:*** 13, 9, 10; ***E4:*** 10, 13, 13; ***E5:*** 15, 4, 15
**Line profile analysis of AZ-specific protein distribution at the calyx of Held (Fig. 3h, j; Fig. S13e-o)**			
All analyzed targets	see above: 'Line profile analysis of the overall protein distribution'	***E1:*** 2, ***E2:*** 3, ***E3:*** 3, ***E4:*** 3, ***E5:*** 2 calyces	2 AZ-positive & 2 AZ-negative ROIs per calyx; for available experiments see above: 'Line profile analysis of the overall protein distribution'
**Colocalization analysis of AZ-specific protein distribution at the calyx of Held (Fig. 3m; Fig. S14)**			
All analyzed targets	see above: 'Line profile analysis of AZ-specific protein distribution'	***E1:*** 2, ***E2:*** 3, ***E3:*** 3, ***E4:*** 3, ***E5:*** 2 calyces	2 AZ-positive & 2-AZ-negative ROIs per calyx; for available experiments see above: 'Line profile analysis AZ-specific protein distribution'
**Control experiment: treatment with three different elution buffers (Fig. S5a)**			
$MgCl_2$ condition	1	5/5 U2OS cells	Full-frame analysis
SDS condition	1	6/6 U2OS cells	Full-frame analysis
GdnHCl condition	1	8/8 U2OS cells	Full-frame analysis
**Control experiment: three different incubation times for elution (Fig. S5b)**			
5 min condition	1	6/6 U2OS cells	Full-frame analysis
15 min condition	1	6/6 U2OS cells	Full-frame analysis
25 min condition	1	5/5 U2OS cells	Full-frame analysis
**Control experiment: four different bleaching times Fig. S5c)**			
All conditions	1	3/3 U2OS cells	Full-frame analysis
**Control experiment: cross-talk with Tom20 as test case (Fig. S6a)**			
All data sets (1-6)	3	3/9 U2OS cells	3 ROIs in Tom20-positive area per cell, 3 ROIs in cell area with background signal
**Control experiments: elution and bleaching with multiple targets as test case (Fig. S8b-d)**			
All analyzed targets	1	3/3 U2OS cells	3 ROIs in specifically labeled area per cell, 3 ROIs in cell area with background signal
**Control experiments: repeated bleaching and elution in U2OS cells (Fig. S9a, e)**			
All analyzed targets	1	6/6 U2OS cells	Full-frame analysis
**Control experiments: repeated bleaching and elution in MNTB tissue (Fig. S9c, f)**			
All analyzed targets	1	7/7 fields of view	Full-frame analysis
**Localization precision and spatial resolution (Fig. S10a, b, c)**			
All data sets	1	1/1 U2OS cell	1 ROI per staining round and channel
**Nuclear pore complex metrics (Fig. S10c)**			
All analyzed targets	1	1/1 U2OS cell	10 nuclear pore complexes

Fig. SXy = Supplementary Figure Xy, MNTB = medial nucleus of the trapezoid body
