# Supplementary material for: Automated highly multiplexed super-resolution imaging of protein nano-architecture in cells and tissues
Source: Nat Commun. 2020 Mar 25;11:1552. doi: 10.1038/s41467-020-15362-1 (PMC7096454; doi:10.1038/s41467-020-15362-1)
Supplement: Supplementary file 3 — Description of Additional Supplementary Files [file 41467_2020_15362_MOESM3_ESM.pdf]

Description of Additional Supplementary Files

File Name: Supplementary Movie 1
Description: **Performance of the maS^{3}TORM setup.** Video demonstrating buffer preparation, liquid exchange, approaching sample regions and image acquisition by maS^{3}TORM.

File Name: Supplementary Movie 2
Description: **Super-resolution images of three target proteins from different imaging rounds merged in 3D space.** STORM images of phalloidin (red), α-tubulin (green), and Tom20 (blue) rendered and merged in 3D
