# Supplementary material for: Automated highly multiplexed super-resolution imaging of protein nano-architecture in cells and tissues
Source: Nat Commun. 2020 Mar 25;11:1552. doi: 10.1038/s41467-020-15362-1 (PMC7096454; doi:10.1038/s41467-020-15362-1)
Supplement: Supplementary file 6 — Reporting Summary [file 41467_2020_15362_MOESM6_ESM.pdf]

nature research

Corresponding author(s): *Double-blind peer review submissions: write DBPR and your manuscript number here* Thomas Kuner

Last updated by author(s): Feb 19, 2020

Reporting Summary

Nature Research wishes to improve the reproducibility of the work that we publish. This form provides structure for consistency and transparency in reporting. For further information on Nature Research policies, see Authors & Referees and the Editorial Policy Checklist.

Statistics

For all statistical analyses, confirm that the following items are present in the figure legend, table legend, main text, or Methods section.

n/a	Confirmed	
☐	☒	The exact sample size (*n*) for each experimental group/condition, given as a discrete number and unit of measurement
☐	☒	A statement on whether measurements were taken from distinct samples or whether the same sample was measured repeatedly
☐	☒	The statistical test(s) used AND whether they are one- or two-sided<br>*Only common tests should be described solely by name; describe more complex techniques in the Methods section.*
☐	☒	A description of all covariates tested
☒	☐	A description of any assumptions or corrections, such as tests of normality and adjustment for multiple comparisons
☐	☒	A full description of the statistical parameters including central tendency (e.g. means) or other basic estimates (e.g. regression coefficient) AND variation (e.g. standard deviation) or associated estimates of uncertainty (e.g. confidence intervals)
☐	☒	For null hypothesis testing, the test statistic (e.g. *F*, *t*, *r*) with confidence intervals, effect sizes, degrees of freedom and *P* value noted<br>*Give P values as exact values whenever suitable.*
☒	☐	For Bayesian analysis, information on the choice of priors and Markov chain Monte Carlo settings
☒	☐	For hierarchical and complex designs, identification of the appropriate level for tests and full reporting of outcomes
☐	☒	Estimates of effect sizes (e.g. Cohen's *d*, Pearson's *r*), indicating how they were calculated

Our web collection on statistics for biologists contains articles on many of the points above.

Software and code

Policy information about availability of computer code

Data collection	ImageJ 1.50b (open source), µManager 1.4.22 (open source), Microscope Control (custom code: C:\Users\3dstorm\git \MicroscopyControl2), Experiment Editor (custom code: C:\Users\3dstorm\git\MicroscopyControl2), Chronos 4.8.0 (commercial)
Data analysis	rapidSTORM 3 (open source), Post Processing Software (custom code), ImageJ 1.50b (open source) with custom-written plugins, Matlab R2018b (commercial) with custom-written scripts, Excel 2013 (commercial), GraphPad Prism 5 (commercial), ViSP v1.0 (open source)

For manuscripts utilizing custom algorithms or software that are central to the research but not yet described in published literature, software must be made available to editors/reviewers. We strongly encourage code deposition in a community repository (e.g. GitHub). See the Nature Research guidelines for submitting code & software for further information.

Data

Policy information about availability of data

All manuscripts must include a data availability statement. This statement should provide the following information, where applicable:

- Accession codes, unique identifiers, or web links for publicly available datasets
- A list of figures that have associated raw data
- A description of any restrictions on data availability

All raw and processed data will be made available upon request.

Field-specific reporting

Please select the one below that is the best fit for your research. If you are not sure, read the appropriate sections before making your selection.

☒ Life sciences ☐ Behavioural & social sciences ☐ Ecological, evolutionary & environmental sciences

Life sciences study design

All studies must disclose on these points even when the disclosure is negative.

Sample size	The multiplex experiment on U2OS cells was designed to prove the feasability of the re-STORM approach. Here, no sample size determination was done. Multiplex experiments on tissue samples were used to analyse protein distribution. Therefore, quantifications were performed on 3 calyces of Held per experiment with at least 3 experiments (up to 5 experiments). For the analysis of the overall synaptic distribution, data were collected from 4-16 line profiles per calyx. For the AZ-specific analysis, 2 AZ-positive and 2-AZ-negative regions were analyzed per calyx. For detailed sample numbers, see Supplementary Table 5.
Data exclusions	No data from samples that passed our requirement were excluded from analyses.
Replication	Every antibody and other labeling agent used for our multiplex experiments was successfully and reproducibly pretested in individual experiments.
Randomization	All calyx segments that passed our demands for a contigous membrane and AZ staining were analyzed equally, and thus, there was no requirement for randomization.
Blinding	Acquisition and quantification were performed fully automatically. Therefore, there was no need for blinding.

Reporting for specific materials, systems and methods

We require information from authors about some types of materials, experimental systems and methods used in many studies. Here, indicate whether each material, system or method listed is relevant to your study. If you are not sure if a list item applies to your research, read the appropriate section before selecting a response.

Materials & experimental systems

n/a	Involved in the study	
☐	☒	Antibodies
☐	☒	Eukaryotic cell lines
☒	☐	Palaeontology
☐	☒	Animals and other organisms
☒	☐	Human research participants
☒	☐	Clinical data

Methods

n/a	Involved in the study	
☒	☐	ChIP-seq
☒	☐	Flow cytometry
☒	☐	MRI-based neuroimaging

Antibodies

Antibodies used	See Supplementary Table 3.
Validation	See Supplementary Table 3.

Eukaryotic cell lines

Policy information about cell lines

Cell line source(s)	The U2OS (human) cell-line were obtained from M. Heilemann laboratory; Initially from CLS Cell Line. Services, Eppelheim, Germany
Authentication	U2OS cells were not authentified. As constititutive expression of Nup133-Ypet was present in all cells observed, post-hoc contamination by other cell lines can be excluded.
Mycoplasma contamination	U2OS cells were tested negative for mycoplasma contamination at the source laboratory.
Commonly misidentified lines (See ICLAC register)	No commonly misidentified cell lines were used.

Animals and other organisms

Policy information about studies involving animals; ARRIVE guidelines recommended for reporting animal research

Laboratory animals	Sprague-Dawley wild-type rats, Charles River, 12 days old, sex was not determined.
Wild animals	This study did not involve wild animals.

Field-collected samples	This study did not involve field-collected samples.
Ethics oversight	All experiments were conducted in accordance with the European and German animal welfare guidelines, and approved by the Regierungspraesidium Karlsruhe (protocol G-75/15).

Note that full information on the approval of the study protocol must also be provided in the manuscript.
